# The Exercising Brain: Changes in Functional Connectivity Induced by an Integrated Multimodal Cognitive and Whole-Body Coordination Training

**DOI:** 10.1155/2016/8240894

**Published:** 2015-12-27

**Authors:** Traute Demirakca, Vita Cardinale, Sven Dehn, Matthias Ruf, Gabriele Ende

**Affiliations:** Department of Neuroimaging, Central Institute of Mental Health, Medical Faculty Mannheim, Heidelberg University, J5, 68159 Mannheim, Germany

## Abstract

This study investigated the impact of “life kinetik” training on brain plasticity in terms of an increased functional connectivity during resting-state functional magnetic resonance imaging (rs-fMRI). The training is an integrated multimodal training that combines motor and cognitive aspects and challenges the brain by introducing new and unfamiliar coordinative tasks. Twenty-one subjects completed at least 11 one-hour-per-week “life kinetik” training sessions in 13 weeks as well as before and after rs-fMRI scans. Additionally, 11 control subjects with 2 rs-fMRI scans were included. The CONN toolbox was used to conduct several seed-to-voxel analyses. We searched for functional connectivity increases between brain regions expected to be involved in the exercises. Connections to brain regions representing parts of the default mode network, such as medial frontal cortex and posterior cingulate cortex, did not change. Significant connectivity alterations occurred between the visual cortex and parts of the superior parietal area (BA7). Premotor area and cingulate gyrus were also affected. We can conclude that the constant challenge of unfamiliar combinations of coordination tasks, combined with visual perception and working memory demands, seems to induce brain plasticity expressed in enhanced connectivity strength of brain regions due to coactivation.

## 1. Introduction

Already in 1949 Hebb proposed that simultaneous neuronal firing stimulates synaptic plasticity [1]. Later several studies found evidence for experience-dependent neurogenesis in the hippocampi of adult mice (for a review see [2]). Today there is accumulating evidence that also the human brain continues to be shaped by experience throughout adulthood [3–5]. These adaptive changes have been shown to take place on structural as well as functional level [6–9].

A practicable approach to study experience-dependent plasticity in humans is to investigate longitudinal changes in brain structure or function following exposure to training. Recently, a number of studies have been published that investigated the effect of training on the functional architecture of the brain by resting-state fMRI (rs-fMRI) (for a review see [9, 10]). Resting-state functional connectivity is commonly defined as temporal correlations of spontaneous, low frequency fluctuations of the BOLD signal between brain areas during rest due to common history of coactivation. As such, it allows a task-independent assessment of training-related changes in brain function [11–14].

Training studies can roughly be subdivided into motor and cognitive interventions. Motor training varied from joystick tracking tasks [15], chopstick handling [16], finger tapping [17], and force-field learning [18] to whole-body balancing [19] and aerobic fitness training [20]. Training duration varied from 11 minutes [15] to several weeks or months [20]. In the cognitive domain training comprised working memory training [21, 22], multitasking [23], and logical reasoning [24] and duration varied from 4 weeks to 3 months.

Newer approaches used also video-gaming [25] and fMRI based neurofeedback [26, 27].

Within the motor domain, several research groups investigated different kinds of motor skills training with varying duration, intensity, and complexity. Overall, changes in intrinsic functional connectivity were located in sensorimotor and cerebellar areas. In these areas both intrinsic functional connectivity increases [15, 17–19] and decreases [16–19] have been found; decreases were rather associated with cerebellar regions [16, 18]. In the studies conducted by Taubert et al. [19] and Ma et al. [17] intrinsic functional connectivity decreased back to baseline whereas decreases were found by the groups of Yoo et al. [16] and Vahdat et al. [18]; there, the intrinsic functional connectivity after the training was reduced compared to before the intervention.

In the cognitive domain, training rather affected intrinsic functional connectivity between frontal and parietal areas [21, 22, 24]. However, the precise location of training-related change in intrinsic functional connectivity differs between studies. Regarding the variety of changes found by different research groups, the training effects seem to be rather specific to the content of the training, the duration, the intensity, and the timing of the resting-state quantification. However, the studies mentioned before show that changes in intrinsic functional connectivity can reliably be induced by training, that is, experience, across a variety of domains.

The majority of published intervention studies investigated the effect of unimodal training. Within the field of healthy aging research the question rises if combined interventions might be more successful than unimodal interventions (for a review see [28]). Also in the context of studies investigating effects of physical exercise on neuroplasticity and cognition it is suggested that adding cognitive training might enhance the beneficial effect of physical training (for a review see [29]). Yet, there are only few studies that focus on the effect of combined interventions. To our knowledge, there are only two studies exploring the effect of a multimodal training using neuroimaging techniques [30, 31]. In both studies, physical and cognitive training were performed apart from each other. In Li et al.'s study older adults took part in tai chi exercises at one time and memory training and supportive group counselling on another time. They found increased resting-state connectivity between the medial prefrontal cortex and the medial temporal lobe. In Holzschneider et al.'s study participants engaged in cycling sessions and additional spatial memory training sessions. However, only task-based fMRI changes were quantified. After combined training, changes in brain activation and changes in cardiovascular fitness correlated positively in the medial frontal gyrus and the cuneus.

Here, we investigated the effect of combined whole-body motor coordination training with integrated cognitive exercises in healthy adults. Lutz and colleagues (“life kinetik”: http://www.lifekinetik.de/) developed a multimodal training that combines coordinative, cognitive, and visual tasks in a way that the physical exercise is performed while participants are cognitively challenged at the same time. The training consists of combinations of motor activity and cognitive challenges and the training of visual perception, especially the perception of the peripheral visual field. Moving limbs in different unusual combinations, catching, and throwing objects, thus training the visual perception and limb-eye coordination, is a basic characteristic of the training. Moreover, the training tasks are not practiced to perfection but are modified after a few minutes or whenever the performance reaches about 60%. In addition to the avoidance of boredom and frustration, this is supposed to stimulate the brain to constantly adapt to new unfamiliar challenges. Our motivation was to test a training concept that is flexible and interesting for the participants and includes cognitive and motor elements. Although the “life kinetik” training was originally designed to train the coordination of athletes (soccer players, skiers) the difficulty of the task can easily be adapted to the capabilities of patient populations.

Based on the assumption that spontaneous activity reflects the history of coactivation within a local brain network or between brain regions [26, 32] we expect increases in resting-state connectivity of those brain regions probably involved in the exercises and tasks.

The* thalamus* is a subcortical brain area processing and integrating neocortical inputs and outputs [33]. Its connections seem to decrease with age [34] and diminished in mild cognitive impairment (MCI) and Alzheimer's disease (AD) [35, 36]. It serves as a “switchboard of information” or relay station for sensory information. As the training includes unusual pattern of motoric activity in combination with cognitive task, we expect the connectivity of the thalamus to increase.

All the exercises and tasks involve some motor action; hence we expect changes in the* primary motor area* (BA4, M1) and the* premotor area* (BA6) because not only the execution but also the constant alertness to perform an action is involved in the task. In particular the connectivity to the right motor areas may be increased because the exercises include a considerable amount of movement of the left limbs, which is challenging for the right handed participants.

The* cerebellum* is highly involved in motor activity and learning and the functional connections reflect the connections of the cortex [37, 38] so we can expect some changes in its connectivity as well.

The* frontal eye field (FEF)*, a brain region responsible for eye movement and gaze control, is known to be altered in the course of learning to handle moving objects [39–41], which is also part of the exercise, except that this is not trained to perfection like in juggling.

The whole* visual cortex* is additionally challenged by the attempt to train the peripheral vision and the manipulation of different moving objects and due to the possibility of assigning the requested action via a visual stimulus (specific gesture of the trainer or colours). So the primary as well as the secondary visual cortices (BA17, BA18, and BA19) are expected to change their connection to other brain regions.

Each exercise or task consists of chains of movements, which alternate randomly. The prompt to change is frequently given by a verbal command. Hence, we expect an increased connectivity between* auditory areas* (primary and secondary BA41 and BA42) and other brain regions especially the motor and premotor area and as a result of repeated coactivation.

The functional connectivity from and to the* dorsolateral prefrontal cortex* (DLPFC) may be increased because the working memory is involved in linking the action or movement to the assigned command or prompt. The* anterior cingulate cortex* (ACC) is also expected to be involved as a region needed for error detection and impulse control and might accordingly change the connection to other brain regions.

## 2. Methods

### 2.1. Participants

32 right handed subjects with no history of psychiatric or neurological illness were included in the study. 21 individuals (12 females, mean age 48 (±9) years) participated in eleven or twelve of the 13 “life kinetik” training sessions (1 hour per week). The other 11 subjects (7 females, mean age 49 (±8) years) were interested in the training but were not able to attend due to their time schedule but completed two MRI scans.

The study was approved by the Ethics Committee of the Medical Faculty Mannheim, University of Heidelberg, and performed in accordance with the Declaration of Helsinki.

### 2.2. Training Description

“Life kinetik” training pursues the goal to combine motor coordination exercises with cognitive training with an emphasis on working memory. The motor coordination exercises can involve multiple limbs at the same time. Additionally, most of the time one or more pieces of sports equipment (e.g., ball, racket, juggling balls, and scarves) are used which have to be thrown, caught, bounced, or similarly manipulated. The cognitive aspect comes into play by assigning distinct motor tasks to different visual or auditory cues (symbols/key words). For example, a red flag might indicate bouncing a ball with the left hand while a blue flag indicates throwing and catching a ball with the right hand. The same movement-cue coupling can be done with semantic categories, for example, city names, animals, or trees. These pairs of motor task and specific cue have to be memorized during one training session. The randomization of cues is self-evident. Within one training session (1 hour per week) approximately 6 different types of exercises have been performed either in groups, in pairs, or by oneself.

An essential aspect of this combined training is that the exercises are not trained until automatized. As soon as participant's performance reaches about 60% correct trials the task demands are changed and new combinations of symbols and movements are introduced. The focus on novelty is supposed to constantly challenge the participants. Moreover, cross talk of the hemispheres is fostered by including movements where limbs purposefully cross the sagittal midline (e.g., to catch a ball arriving at the left side of the body with the right hand).

In total, there were 13 training sessions of 1 hour per week of which our participants followed at least 11; that is, the minimal training duration was 11 hours across a period of 13 weeks.

### 2.3. Data Acquisition

Functional and anatomical data were acquired from each participant within 2 weeks before the start of the first training session and within 2 weeks after the last training session on a 3 Tesla MRI Scanner (Magnetom Trio with TIM technology, Siemens Medical Service, Erlangen, Germany) equipped with a 32-channel head coil. 200 rs-fMRI images were acquired with gradient echo T2^*∗*^-weighted echo-planar-imaging sequence (TE = 28 ms, TR = 1.79 s, FOV = 192 mm × 192 mm, matrix size = 64 × 64, and total duration is 6 min). A volume comprised 34 slices in AC-PC orientation with a thickness of 3 mm and slice gap of 1 mm. Participants' heads were lightly restrained using soft pads to prevent head movement. Subjects were instructed to look at the fixation cross and keep their eyes open.

A T1-weighted anatomical image was also recorded (TE = 3.03 ms, TR = 2.3 s, 192 slices and FOV = 256 mm × 256 mm, matrix size is 256 × 256, and slice thickness is 1 mm).

### 2.4. Data Preprocessing

Data were preprocessed and analyzed using SPM12 (The Wellcome Department of Cognitive Neurology, London, UK, http://www.fil.ion.ucl.ac.uk/spm/software/spm12/). All functional images were slice-time corrected and realigned to the first volume using a six-parameter rigid body transformation. Threshold for exclusion due to excessive motion was set to 3 mm. The movement was not more than 1.5 mm in each subject, so no one had to be removed.

The anatomical image and functional images were coregistered for the corresponding time-point. Segmented gray matter and white matter images of all participants were used to construct a study specific template using DARTEL [42]. The template was normalized to MNI space and all images, anatomical and functional, were normalized to this template using the according flow fields. The smoothing kernel for the functional images was 8 mm and 2 mm for the anatomical image.

### 2.5. Connectivity Analysis

Functional connectivity analyses were carried out using the CONN-fMRI functional connectivity toolbox v14 [43] (http://www.nitrc.org/projects/conn). The modest test-retest reliability of the rs-fMRI seems attributable to remaining noise after preprocessing, adding nonneural correlation to the BOLD signal [44]. Removing the noise is a possibility to increase the reliability of rs-fMRI data. Several preprocessing steps have been proposed [44] to achieve this.

One major point is reducing the noise via the anatomical CompCor approach. This method extracts principal components (5 each) from WM and CSF time series. WM and CSF voxels are identified via a segmentation of the anatomical images. These components are added as confounds in the denoising step of the CONN toolbox [43, 45]. The six head motion parameters derived from spatial motion correction were also added as confounds. We did not perform global signal regression as the discussion about the impact is still ongoing [44, 46] and it is not available on the CONN toolbox.

As recommended band-pass filtering was performed with a frequency window of 0.01 to 0.1 Hz. This preprocessing step was found to increase the retest reliability [44].

Seed-to-voxel and ROI-to-ROI functional connectivity maps were created for each participant. The ROI-to-ROI analysis was used to identify possible differences between trainees and control subjects at pretraining and to verify that brain networks of control subjects did not change over time. For this analysis we used all the provided Brodmann areas. The mean BOLD time series was computed across all voxels within each ROI. Bivariate-correlation analyses were used to determine the linear association of the BOLD time series between each pair of sources and a Fisher Z transformation was applied.

Individual seed-to-voxel and ROI-to-ROI maps were entered into a second-level analysis.

A within group ROI-to-ROI analysis for the control group tested the stability of the connectivity over time. A between groups ROI-to-ROI analysis verified the lack of differences between the two groups for the first measurement.

Seed-to-voxel analyses were used for two purposes. First we used the posterior cingulate and the medial prefrontal cortex as seed region and verified the occurrence of the default mode network in each group and to both time-points. This seems necessary due to the different size of the two groups.

The second seed-to-voxel analysis was used to examine differences in connectivity changes in a 2 × 2 factorial analysis with time by training interaction (group *∗* time; contrast −1 +1 +1 −1). Age and sex were entered as covariates of no interest in the analysis [47]. The threshold for significant changes was set to *p* = 0.05 whole brain cluster level FWE corrected with a cluster building threshold of *p* = 0.001 uncorrected on voxel level. As we expected an increase in training participants due to coactivation and no change in control subjects we verified the direction of changes with two post hoc paired sample *t*-tests for the trainee and the control group separately for each significant seed-to-voxel cluster. This step was chosen to assure that the significant results were not caused by between-subject variance. The other reason for this approach was the different sample size of the two groups. We report significant results due to three criteria: (a) significant time by group interaction, (b) significant increase within the training group, and (c) no significant decrease in the control group.

For display purposes the cluster building threshold for the result-figure was set to 0.002 uncorrected on voxel level.

### 2.6. Regions of Interest

As we could not investigate task related activity for the exercises and it is somewhat arbitrary and prone to bias to create a region of interest out of a single coordinate and an according sphere, we used the provided ROIs that are based on the Brodmann areas according to the WFU PickAtlas (http://fmri.wfubmc.edu/software/PickAtlas). We used all existing areas as ROIs, in order to get a complete picture of possible changes within the control group. Some ROIs are not provided by the toolbox (e.g., thalamus, cerebellum, and FEF); here we created ROI using the masks provided by WFU PickAtlas of the according brain region.

## 3. Results

ROI-to-ROI analysis for the rs-fMRI at the first time-point showed no differences between trainees and controls. The default network could be shown with the medial prefrontal cortex as seed in both groups and both time-points.

The impact of the training was analyzed by a 2 × 2 ANOVA (group and time) with age and sex as covariates of no interest. All seed regions with significant positive connectivity changes in trainees and no significant decreases in controls are listed in Table 1.

**Figure 1:**
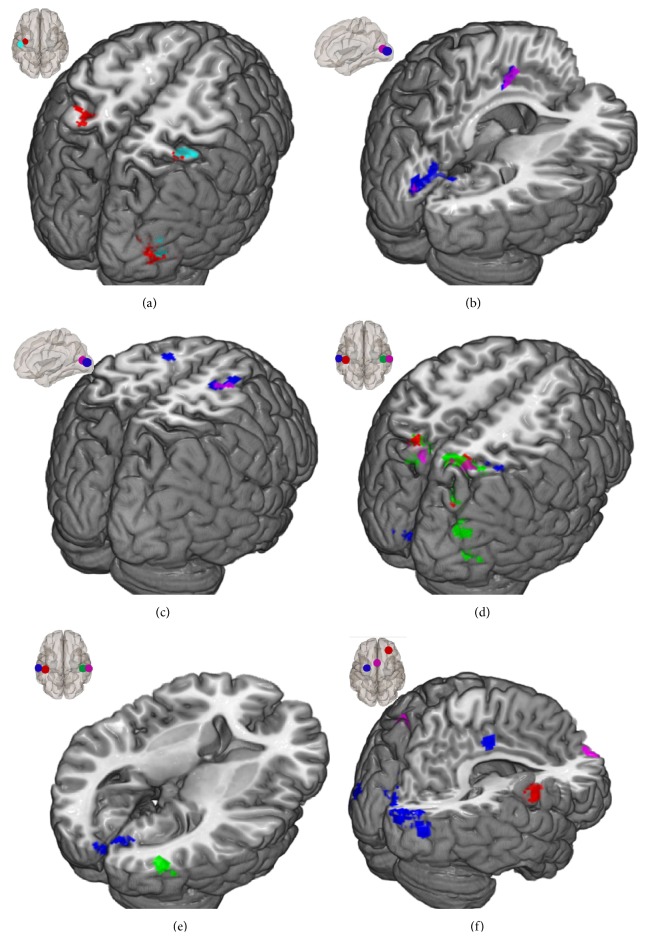
Greater connectivity increases in trainees compared to controls subjects; seed regions are represented in the small brain images; seeds determine the colour of the result region: (a) BA4 left: red and BA1 left: cyan; (b) and (c) visual cortex: BA17 right: violet and BA18 right: blue; (d) and (e) auditory cortex: BA41 right: green, BA41 left: red, BA42 right: violet, and BA42 left: blue; (f) dorsolateral prefrontal cortex: right: red, FEF left: blue, and midcingulate cortex: violet.

The training involved a great amount of motor activity and the motor region was one of the hypothesized regions changing their connectivity strength. The increase occurred only for* the left motor region*. The left primary motor area (BA4, M1) showed increased connectivity to parts of the visual cortex (Figure 1(a), red) and the somatosensory association area (BA7, Figure 1(a), red). The right primary motor cortex showed no changes to any other brain region. The connectivity strength of the whole* premotor areas* (BA6) as seed to other cortical regions did not change.

The primary* sensorimotor cortices* (BA1, BA2, and BA3) as part of the sensorimotor network showed few changes in connectivity strength. Only the spontaneous fluctuations of the left BA1 showed higher correlation to parts of the associative visual cortex (BA19, Figure 1(a), cyan) and parts of the parietal cortex (BA7, Figure 1(a), cyan). Connectivity from BA2 or BA3 did not change.

The functional coupling within the visual network changed for the* primary sensory areas* (BA17) of the right hemisphere. This ROI increased in functional connectivity to the ventral ACC (BA24, Figure 1(b), violet) and parts of the right premotor cortex (BA6, Figure 1(c), violet). The connection to the left premotor cortex (Figure 1(c), blue) was increased for the right* secondary visual cortices* (BA18). The connection increase to the ventral ACC (midcingulate; Figure 1(b), violet and blue) of the visual areas was overlapping. Different areas of the visual cortex show changes in functional coupling to the same premotor region and the cingulate cortex.

The functional connectivity strength between* the primary auditory cortex* (BA41) as part of the auditory network and the right cerebellum (areas VIII and IX) increased with the training as well as the connection to the somatosensory association cortex (BA7, Figure 1(d), green and red). This connectivity change was interhemispheric and overlapping. The connections of the* secondary auditory cortex* (BA42) to the parietal cortex (BA7, Figure 1(d), violet and blue) changed as well, partly overlapping with the increased connectivity of the primary auditory cortex. Connections from the auditory to the visual cortex did also increase (Figures 1(d) and 1(e), blue and green).

The* left FEF* but not the right FEF showed connectivity changes to several clusters in the visual cortex (Figure 1(f), blue) and the ventral ACC (Figure 1(f), blue).

The connectivity between* the right dorsolateral prefrontal cortex* and the right supramarginal gyrus (Figure 1(f), red) increased. The ACC (BA24) showed no increase in connectivity. A more posterior part* of the cingulate gyrus* showed an increased functional connectivity to the right anterior frontal cortex and partially of the dorsolateral prefrontal cortex (BA10 and BA9, Figure 1(f), violet).

The characteristic regions of the default mode network,* medial prefrontal cortex*,* lateral parietal cortex*,* posterior cingulate*, and* superior frontal cortex,* showed no change in connectivity after the training.

## 4. Discussion

This study is the first to investigate the impact of an integrated multimodal training on functional brain connectivity. The training combines physical and cognitive exercises and does not aim at automating but focuses on novelty. For this purpose we compared the changes of the resting-state intrinsic functional connectivity of a group of subjects attending the first standard training course of “life kinetik” with the rs-fMRI changes of a control sample. The training offers a great variety of exercises and can easily be adapted to clinical populations. We found a considerable amount of changes in resting-state functional connectivity in the training group. The connections within the default mode network, the most prominent resting-state network, did not change.

The changes in functional connectivity mirror the activation during the training and some increases in correlation occur in regions that are known to be a key region in cognitive deficits, ageing or mental illness.

Connectivity increases of the motor region were assumed to be the most probable ones. In particular the connections from the right motor and premotor cortex, responsible for the left part of the body, were expected to strengthen. The training involved a great amount of motor activity and all participants were right handed, thus being forced to coordinate their left hand, arm, and leg. An increase of the connectivity for the whole region was only visible for the left primary motor area. The connection to parts of the somatosensory association area changed as well as to the visual cortex. The involvement of motor areas in brain plasticity has been corroborated in several studies. Musicians, for example, who have a long history of motoric training showed increased resting-state connectivity in motor areas and multisensory cortices compared to a control group [48]. This is in contrast to other studies that found a decrease in connectivity accompanied with cumulative performance increase after initial “beginners” increase of connectivity [17]. As the “life kinetik” training focuses on the novelty of the exercises a decrease of connectivity was not expected.

Various seed regions in the visual cortex showed an increased connectivity with parts of the premotor cortex, almost overlapping (see Figure 1(c)). A great number of the training tasks consisted of throwing and catching different and, in some cases, relative small objects. The most comparable task used in other studies was juggling training where an impact of training intensity was found [49]. Low intensity training resulted in increases in functional connectivity in the motor network, whereas the high intensity juggling training group showed decreased functional connectivity. The results suggest that different training regimes are associated with distinct patterns of brain change [50]. Our training on the other hand was much less intensive than the low intensity juggling training which consisted of 15 min per day and furthermore, as already mentioned, “life kinetik” training is not directed to perfection.

The cerebellum is mapped to the association areas of the cerebrum [37] so that we expected some changes in connectivity, which we found but less pronounced than expected. In contrast to the analysis of Buckner et al. [37] who found that the primary sensory cortices were not represented in the cerebellum, we found a change in the correlation of time courses in the primary auditory cortex and the cerebellum. Maybe this is an effect of the verbal prompts during the exercises, which indicated different movements.

The visual cortex is also diversified. Some subregions responsible for the retention of visual-motion information [51] were shown to change their structure during motor training. Not structural but functional changes occur in our sample of trainees in contrast to the control sample.

The brain region with the most prominent connectivity changes was the secondary somatosensory association cortex (BA7). Several parts of BA7 exhibited connectivity increases with other regions. Mainly the auditory cortices change their relation to parts of BA7 (Figure 1(d)). These clusters are all overlapping irrespective of the according seed region. Also distinct from these regions are the clusters changing the connections to the motor cortex (Figure 1(b), red) and the sensorimotor cortex. This result seems to corroborate the findings of the diversification of the parietal cortex [52, 53]. Grasping and visuospatial tasks activated different parts of the parietal cortex [53], overlapping with the regions that showed changes during “life kinetik” training (Figures 1(a) and 1(d)).

The functional connectivity between the left FEF as seed region and clusters in the visual cortex and the ventral ACC increased, but not with the dorsal attention network. The FEF is responsible for eye movement and surely active during throwing and catching of objects. The change of connectivity between FEF and visual cortex might be an indication for a combined activation of these regions due to the increased visual attention during the training.

Regions of the frontal cortex involved in working memory processes and error processing showed few connectivity increases. The ACC showed, in contrast to our hypothesis, no changes in connectivity to other brain regions. The ventral part of the cingulate cortex showed increased connectivity to the anterior prefrontal cortex, to the FEF, and to the visual cortex. The left dorsolateral prefrontal cortex (BA9) on the other hand showed an increased connectivity to the right supramarginal gyrus (BA40).

Given the specific property of the training, the connectivity changes seem reasonable. Prompts for the movements and tasks are given by verbal or visual cues. The cue has to be translated to an action, in most cases movements or manipulation of objects. Parts of the premotor regions showed increased connections to visual areas. These regions were not distinct but overlapping thus indicating the importance of these regions for preparing the action and also for the object manipulation [54].

The question is why the auditory areas predominantly showed increased functional connectivity to the somatosensory areas (BA7) but not to the premotor area. Attention is one important aspect of the training in combination with working memory. One major effort for the trainees is to remember the according movement to the prompt. But this did not result in the expected changes in connectivity of the dorsolateral prefrontal cortex.

The functional connection of the thalamus to the right inferior frontal gyrus and insula increased in trainees but also decreased in controls. This makes the interpretation difficult. The thalamus is a region with multiple connections [33, 55, 56]. The strength of the thalamocortical connection has been reported to predict the performance in motor learning [57], to change with age [34], and to be diminished in MCI and AD [35, 36], and a disruption of the thalamus-cortex relation has severe implications on mental health [58–60].

Proper or optimal function of BA7 seems to be an indication for a cognitive reserve, preventing dementia symptoms [61]. Switching attention is an important part of the training; regions that are activated in such a task are part of the parietal cortex as well as premotor areas and the dorsolateral PFC [62] but the relation between these regions and especially the change in relation have not yet been investigated.

With our study we could show that the applied “life kinetik” training changes the connectivity strength between several brain regions. There is a lot of evidence for brain plasticity even in the adult and aging brain. Basic research has shown that different aspects of the brain can be shaped by various types of training and tasks. Resting-state connectivity seems to be relatively stable [63], but disturbed in psychiatric disorders [64], changing with age [65, 66], and changeable by activity [26]. Intrinsic connectivity is shown to be an indicator for efficiency [67] and positively correlated with cognitive performance [68, 69] and intelligence [70].

The data on the direction of changes or alterations in terms of increase or decrease are inconsistent. It is not generally known which direction is more beneficial. This most likely depends on many functional aspects the connectivity is supporting. For example, patients with major depression show an increased functional connectivity [71] whereas schizophrenia seems to be accompanied by decreased functional connectivity [60].

The functional connectivity of the motor cortex, for example, is increasing with age but different relations to performance were reported. One study interpreted the positive relation of connectivity and performance as a protection against decline [66]. The second study found the increase in connectivity with age accompanied by poorer performance [65]. This contradicting consequence of connectivity increase with regard to performance demonstrates that an intervention leading to enhanced connectivity between brain areas might not necessarily help attenuate age related decline.

The tasks and types of training used in different studies to investigate the change of brain networks are somewhat arbitrary, varying from perception tasks according to robot-hand movements to juggling or transcranial electrical or magnetic stimulation. Most training concepts do not have the potential to serve as a training method or therapy approach for psychiatric patients or elderly individuals.

Exceptions are various types of motor training like juggling [50], video games [25], aerobic training [72], or the quadrato-motor training [73] which are all aimed at perfecting the task without varying the task.

Our motivation was to look for a training that includes motor and cognitive exercises and has the potential to be stimulating for a patient population.

### 4.1. Limitations

Test-retest reliability is a not yet completely resolved issue in fMRI studies [74–77]. Few studies addressed this issue for rs-fMRI but retest reliability was found to be robust [44, 78]. Improvement can be made via the inclusion of several preprocessing steps [44]. This enhances the intersession retest reliability to 0.81. We addressed this issue by including preprocessing steps that are known to reduce noise [44, 79, 80]. We carefully screened the control group for changes and reported only results with a significant post hoc test.

A second limitation for our results is the whole sample size as well as the difference in the size of the trainee and control group. We tried to address this issue by verifying the occurrence of the default mode network that did not change in control subjects despite the small sample size.

The impact of the training intensity is unknown. The actual program consisted of 1-hour training per week. Further studies should investigate the effect of shorter but more frequent training sessions.

The subject group participating in the training showed an increase in resting-state functional connectivity but the impact on task performance is unknown and could not be monitored due to the nature of the training. A next step would be to find suitable motor and cognitive test tasks to quantify an improvement following “life kinetik” training.

Further investigations should include an active control group (practicing either motor performance or cognitive tasks or training a limited number of tasks to perfection) to show the benefit of the combined training compared to its isolated parts. Furthermore, we will investigate the impact of the training on cognitive performance and memory, preferably in a group of impaired subjects.

Since it is not possible to measure the brain activity during the training, we only could assume which brain regions are activated. This study was planned as a pilot study to show that the training is able to change brain connectivity. We assume that our subjects show “normal” resting-state networks. In a patient group the connectivity increases may depend on the underlying alteration of the according network.

## Figures and Tables

**Table 1:** Seed-to-voxel results of 2 × 2 ANOVA (*t*1 < *t*2; trainees > controls), age and sex as covariates, cluster building threshold *p* = 0.001 uncorrected, cluster threshold *p* = 0.05 FWE corrected, post hoc test increase in trainees, and no decrease in controls.

Figure/colour	Seed	Seed hemisphere	Result region	BA	Result hemisphere	*x*	*y*	*z*	*k*	Cluster *p*-FWE	Cluster *p*-unc	Peak *p*-FWE	Peak *p*-unc
	*Motor area*												
(a)/red	BA4/M1	Left	Somatosensory association cortex	BA7	Left	−30	−48	52	83	0.04605	0.00103	0.373	0.000002
(a)/red	BA4/M1	Left	Somatosensory association cortex	BA7	Right	26	−44	48	99	0.01981	0.00044	0.982	0.000035
(a)/red	BA4/M1	Left	Associative visual cortex	BA19	Right	38	−78	−4	258	0.00002	0	0.791	0.000011

	*Sensory-motor area*												
(a)/cyan	BA1	Left	Associative visual cortex	BA19	Right	40	−82	8	137	0.00354	0.00008	0.869	0.000015
(a)/cyan	BA1	Left	Somatosensory association cortex	BA7	Right	30	−42	50	126	0.0059	0.00013	0.987	0.00004

	*Visual area*												
(b)/violet	Primary visual cortex BA17	Right	Ventral ACC	BA24	Left	−8	4	38	81	0.04406	0.00095	0.477	0.000003
(c)/violet	Primary visual cortex BA17	Right	Premotor cortex	BA6	Right	22	−8	66	133	0.00294	6.2*E* − 05	0.844	0.000012
(b)/blue	Secondary visual cortex BA18	Right	Secondary visual cortex	BA18	Left	−14	−82	2	329	1*E* − 06	0	0.005	0
	Secondary visual cortex BA18	Right	Dorsal posterior cingulate cortex	BA31	Right	14	−66	20	85	0.03536	0.00076	0.223	0.000001
(b)/blue	Secondary visual cortex BA18	Right	Ventral ACC	BA24	Left	−6	12	40	97	0.01841	0.00039	0.718	0.000008
(c)/blue	Secondary visual cortex BA18	Right	Premotor cortex	BA6	Left	−20	−2	60	96	0.01942	0.00041	0.923	0.000019

	*Auditory area*												
	Primary auditory cortex BA41	Left	Cerebellum	Cerebellum (8 & 9)	Right	20	−34	−46	163	0.00073	1.5*E* − 05	0.003	0
(e)/green	Primary auditory cortex BA41	Right	Somatosensory association cortex & associative visual cortex	BA7 & BA19	Right	36	−78	12	874	0	0	0.007	0
(d)/green	Primary auditory cortex BA41	Right	Somatosensory association cortex	BA7	Left	−26	−54	40	218	4.8*E* − 05	1*E* − 06	0.951	0.000022
(d)/red	Primary auditory cortex BA41	Left	Somatosensory association cortex	BA7	Right	14	−66	52	200	0.00014	3*E* − 06	0.933	0.00002
(d)/red	Primary auditory cortex BA41	Left	Somatosensory association cortex	BA7	Left	−10	−70	52	131	0.00327	6.9*E* − 05	0.932	0.00002
(d)/violet	Secondary auditory cortex BA42	Right	Somatosensory association cortex	BA7	Right	22	−64	54	76	0.04641	0.00094	0.864	0.000012
(d)/violet	Secondary auditory cortex BA42	Right	Somatosensory association cortex	BA7	Left	−20	−58	40	131	0.00225	4.4*E* − 05	0.583	0.000004
(d)/blue	Secondary auditory cortex BA42	Left	Somatosensory association cortex	BA7	Right	34	−50	48	130	0.00319	6.6*E* − 05	0.568	0.000004
(e)/blue	Secondary auditory cortex BA42	Left	Secondary visual cortex	BA18	Midline	0	−92	0	140	0.00196	4.1*E* − 05	0.983	0.000033

	*Frontal cortex *												
(f)/red	Dorsolateral prefrontal cortex (BA9)	Right	Supramarginal gyrus	BA40	Right	64	−18	24	192	0.00026	6*E* − 06	0.737	0.000008
(f)/blue	Frontal eye field/superior frontal	Left	Ventral ACC	BA24	Right	8	−12	38	110	0.00696	0.00014	0.563	0.000004
(f)/blue	Frontal eye field/superior frontal	Left	Secondary visual cortex	BA18	Right	36	−90	6	397	0	0	0.577	0.000004
(f)/blue	Frontal eye field/superior frontal	Left	Secondary visual cortex	BA18	Left	−24	−96	14	193	0.00012	2*E* − 06	0.771	0.000009
(f)/violet	Midcingulate gyrus	Right	Anterior prefrontal cortex & dorsolateral prefrontal cortex	BA10 & BA9	Right	6	66	18	100	0.01336	0.00027	0.934	0.000019

BA = Brodmann area, *x*, *y*, and *z* = MNI coordinates, *k* = cluster size, and (a)–(f) = parts of Figure 1.
